# Y.W. Loke, *Life’s Vital Link: The Astonishing Role of the Placenta*

**DOI:** 10.1093/emph/eou004

**Published:** 2014-02-06

**Authors:** Deena Emera

**Affiliations:** Yale University School of Medicine, 333 Cedar Street SHM I120, New Haven, CT 06510, USA *E-mail: deena.emera@yale.edu*

This fascinating book about the placenta tells the story of this underappreciated yet truly remarkable organ. In the nine chapters of this book (the number is not a coincidence), Y.W. Loke touches on how the placenta evolved, describes its development from conception to birth and details the myriad of complex functions it performs. I highly recommend this book for the layperson (non-scientists and non-reproductive biologists), for its many fascinating facts, up-to-date science and clear explanations.

To explain how and why the human placenta works the way it does, Loke rightfully begins this volume by describing the strategies used by amniotes, non-placental mammals and non-human placental mammals to nourish their young offspring. This all lays the groundwork for understanding how the human placenta evolved and functions. For example, even though the platypus, a monotreme mammal, lays eggs like birds and lizards, the egg spends several weeks in the uterus during which time it is nourished by maternal secretions that diffuse across the eggshell. The production and uptake of these secretions was an important development in the evolution of the placenta, an organ that allows the embryo to obtain nutrients directly from the mother instead of the egg yolk. Loke nicely describes the mammalian placenta in all its diversity, from the superficial epitheliochorial placenta found in ruminants to the deeply invasive hemochorial placenta found in humans. His discussion of placental phylogeny is thorough, up-to-date and clear. The question of which type of placenta evolved first in mammals (superficial or invasive) has been hotly debated over the years, and Loke explains why scientists now think it was invasive, based on the current distribution of placental types among mammals. Later in the book, Loke discusses an exciting hypothesis on how the invasive placenta first evolved: by retroviral infections in our early mammalian ancestors. There is now a great deal of evidence that syncytin genes (and other genes/regulatory regions) of retroviruses were co-opted independently in different placental mammals for their immunosuppressive and fusogenic properties. Thus, it is likely that a retroviral infection in the stem lineage of placental mammals was involved in the emergence of this structure as well. As with other topics in this book, Loke’s discussion of the role of retroviruses in placental evolution is compelling and up-to-date.

In subsequent chapters, Loke provides all the details necessary to understand the form and function of the human placenta. He uses terms such as ‘mop of bucket in blood’ to describe the villous tree of the placenta. This and other analogies help illustrate a complex morphology to non-experts. He describes the intimate and complex relationship between maternal and fetal tissues during pregnancy, and devotes a whole chapter to the ‘tug-of-war’ that occurs at the maternal–fetal interface. Loke clearly explains some aspects of maternal–fetal conflict theory as originally outlined out by David Haig; genomic imprinting in the placenta and brain, and the great diversity of placental types, are manifestations of this conflict at different organizational levels. However, he glosses over other aspects such as the large amount of placental hormones produced during pregnancy. His alternative explanation, that they reflect the health of the placenta, is insufficient. His coverage of reproductive immunology in the chapter ‘Nature’s Transplant’, on the other hand, is thorough, clear and sufficiently detailed to explain the ‘immunological paradox’ of pregnancy and its solution.

While the biological terminology and concepts in this book are advanced, the author does a good job making the material accessible to non-experts. Moreover, there are fascinating details provided in the book that will be of great interest to many. For example, in Loke’s description of antibody transport across the placenta, he discusses Henry VIII’s long string of reproductive failures. It is thought that these failures may have been caused by incompatibility in the Kell blood group between Henry and his wives: since Henry was likely Kell positive, and his wives Kell negative, many of their offspring would have died neonatally from hemolytic disease. In another example, Loke describes the anti-inflammatory properties of the placenta: at the site of implantation in the uterus, something similar to a wound appears without any inflammatory response. Loke goes on to say that women with rheumatoid arthritis have significant improvement of their symptoms when they are pregnant, presumably because of these anti-inflammatory signals produced by the placenta. Interestingly, rheumatoid arthritis itself may be caused by fetal cells that cross the placenta during pregnancy. While traditionally described as an autoimmune disease, it is possible that rheumatoid arthritis and other autoimmune disorders like lupus are actually caused by alloimmune responses to fetal cells that escape the placenta and take up residence in the mother. In support of this idea, autoimmune diseases are found more frequently in older adult women than men.

In addition to these fascinating details, there is a great deal of biomedically relevant information in the book. A recurrent theme is the similarity between placental tissue and cancer, which is intriguing and potentially useful in the search for cancer therapeutics. Loke also discusses common reproductive procedures and technologies in light of placental biology. Up to 4% of all births in Western countries today are conceived by assisted reproductive technologies such as *in vitro* fertilization (IVF). In his discussion of genomic imprinting and epigenetic modification of the placenta, Loke explains why this is cause for concern: placentas of babies conceived by IVF have low levels of DNA methylation, which can result in placental/fetal abnormalities. Another procedure that is commonplace today is the cesarian section. While some cesarian sections are performed to save the life of the baby and/or mother, many are performed out of convenience. There is a clear correlation between prior cesarian section and ‘placenta accreta’, a serious pregnancy complication that can result in maternal hemorrhage. If the placenta of a second pregnancy implants on scar tissue formed after a cesarian section of a first pregnancy, it becomes so firmly embedded in the uterus that it cannot be removed after birth. Loke discusses if this procedure should be discouraged given this connection. As with other issues discussed in the book (e.g. the freezing of umbilical cord blood), Loke provides strong and sometimes self-righteous opinions. Whether or not you agree, the book provides sufficiently detailed and fascinating information on the form and function of the placenta to allow you to come to your own well-informed positions.

## FUNDING

Deena Emera is funded by an NIH NRSA fellowship (Grant #1F32GM106628).

